# Isolation of Prion with BSE Properties from Farmed Goat

**DOI:** 10.3201/eid1712.110333

**Published:** 2011-12

**Authors:** John Spiropoulos, Richard Lockey, Rosemary E. Sallis, Linda A. Terry, Leigh Thorne, Thomas M. Holder, Katy E. Beck, Marion M. Simmons

**Affiliations:** Animal Health and Veterinary Laboratories Agency, Weybridge, Surrey, UK

**Keywords:** zoonoses, bovine spongiform encephalopathy, scrapie, transmissible spongiform encephalopathy, small ruminant, goats, mouse bioassay, prions and related diseases, podcast

## Abstract

Transmissible spongiform encephalopathies are fatal neurodegenerative diseases that include variant Creutzfeldt-Jakob disease in humans, scrapie in small ruminants, and bovine spongiform encephalopathy (BSE) in cattle. Scrapie is not considered a public health risk, but BSE has been linked to variant Creutzfeldt-Jakob disease. Small ruminants are susceptible to BSE, and in 2005 BSE was identified in a farmed goat in France. We confirm another BSE case in a goat in which scrapie was originally diagnosed and retrospectively identified as suspected BSE. The prion strain in this case was further characterized by mouse bioassay after extraction from formaldehyde-fixed brain tissue embedded in paraffin blocks. Our data show that BSE can infect small ruminants under natural conditions and could be misdiagnosed as scrapie. Surveillance should continue so that another outbreak of this zoonotic transmissible spongiform encephalopathy can be prevented and public health safeguarded.

## Abstract 2

BSE can infect small ruminants and could be misdiagnosed as scrapie.

Transmissible spongiform encephalopathies (TSEs) are fatal diseases characterized by neurodegenerative changes in the central nervous system that include vacuolation, gliosis, and accumulation of an abnormal isoform (PrP^Sc^) of a naturally occurring host-encoded protein (PrP^C^) (*1*). According to the prion hypothesis, PrP^Sc^ is the major or the sole infectious agent (*1*). Although this hypothesis has not received universal acceptance, PrP^Sc^ is ubiquitous in all known naturally occurring TSEs, and its detection is widely used for their diagnosis.

Bovine spongiform encephalopathy (BSE), a TSE of cattle, was first detected in 1986 (*2*) and has since been linked with emerging TSEs in other species (*3**,**4*) including humans (*5**,**6*). Because of its ability to cross species barriers and particularly its zoonotic potential, BSE is considered a public health risk, and extensive measures have been established to detect and eliminate the disease.

Scrapie, a naturally occurring TSE affecting small ruminants, has been known for centuries (*7*) and is not considered to pose a public health risk (*8*). Under experimental conditions, however, small ruminants are susceptible to BSE, with pathogenesis and clinical signs that are not readily distinguishable from scrapie (*9**–**12*). Additionally, the fact that small ruminants were exposed to BSE-contaminated food before the exclusion of meat and bone meal from ruminant feedstuffs led to the possibility that sheep and goats on commercial farms could be affected by BSE that could be misdiagnosed as scrapie (*13**,**14*). The response to this potential risk was the implementation of extensive statutory active surveillance, elimination, and breeding for resistance programs in the European Union (EU).

In 2005, as part of a review of historical TSE-positive cases of sheep and goats in France, a specimen from a goat slaughtered for human consumption in 2002 was reported to be “indistinguishable from a BSE isolate on the basis of all identification criteria available.” (*15*). In response to this report, 2 retrospective studies were initiated in the United Kingdom to analyze archived samples from goat cases that were initially diagnosed as scrapie (*16**,**17*). Because only fixed material was available, both studies had to use differential immunohistochemical analysis (D-IHC), a technique that can discriminate scrapie from experimentally induced BSE in sheep (*18*). These studies identified a single case, originally diagnosed in 1990 as scrapie, that had a D-IHC signature indistinguishable from BSE (*16*).

Given the wide phenotypic variance of scrapie in sheep and our limited knowledge of this variance in goats, the D-IHC result on its own was insufficient for an unequivocal diagnosis. In accordance with EU regulation 36/2005 (*19*), the case was referred to the EU Reference Laboratory Strain Typing Expert Group, which recommended further investigation by bioassay.

Bioassay is conventionally undertaken by using unfixed tissues to prepare inocula. Much historical tissue is available only as formalin fixed or formalin fixed and paraffin wax embedded. TSE infectivity persists in such material but with a lower infectious titer than with unfixed frozen tissue (*20*). However, the potential effects on biological activity, and therefore strain characterization, of fixation and processing are unknown. Thus, further investigation of this case required an extensive panel of controls. We report the results of the bioassay analysis and confirm the diagnosis of BSE in a goat in the United Kingdom.

## Materials and Methods

### Sample Preparation

Whenever fixed tissue was used, it had been processed and embedded in paraffin wax. To recover the fixed, paraffin-embedded tissue from the wax blocks, samples were processed in reverse. Specifically, the wax was liquefied by immersing the tissue blocks in a wax bath preheated to 55°C. The samples were then placed in an ether bath and subsequently were immersed in 100% ethanol to remove the ether. This process was followed by sequential washes in alcohol solutions of decreasing concentrations to gradually remove the alcohol and rehydrate the sample. Finally, the samples were suspended in normal saline (10% wt/vol) before homogenization. Unfixed samples were kept frozen at −80°C. After thawing, they were suspended in normal saline (10% wt/vol) before homogenization. All homogenates were examined microbiologically and treated with antimicrobial drugs as required. Only microbiologically cleared inocula were used to challenge animals.

### Experimental Design

The only tissue available from the 1990 suspected UK case (*16*) was paraffin wax–embedded brain (supplied by Martin Jeffrey, Veterinary Laboratories Agency, Lasswade, UK). Several sources were used to control for TSE strain, host species, and tissue condition (i.e., frozen vs. fixed and wax-embedded) to ensure unequivocal interpretation of the results (Table). Paraffin wax–embedded material from 2 field cases that were contemporary with the suspected case but gave a scrapie profile on D-IHC (*16**,**17*) and an experimental caprine BSE case (supplied by Nora Hunter, Roslin Institute, Edinburgh, UK) were used to control for strain variation in material that had been handled and stored in the same way and for a similar time as material from the suspected case (*21*).

**Table:** Bioassay results of first passage (ruminant to mouse) to determine presence of BSE*

Sample source and treatment†	Tissue‡	Mouse line	No. PrP^Sc^-positive mice/total no. mice inoculated§	Clinically positive and H&E-positive mice	Mean (SD) incubation period postinoculation, d
Caprine BSE suspected (V459/90) (*16*)					
Fixed and embedded	Brainstem and cerebellum	C57/BL6	5/20	0	NA
		RIII	5/20	3	525 (93)
		VM	9/20	6	620 (32)
		tg338	8/10	0	NA
Natural caprine scrapie 1 (E90/89) (*16*)					
Fixed and embedded	Brainstem	C57/BL6	0/20	0	NA
		RIII	0/20	0	NA
		VM	0/20	0	NA
		tg338	6/10	0	NA
Natural caprine scrapie 2 (84/1549) (*17*)					
Fixed and embedded	Brainstem	C57/BL6	18/20	18	506 (26)
		RIII	8/20	2	413 (98)
		VM	15/20	6	586 (87)
		tg338	10/10	5	196 (13)
Experimental caprine BSE (45x48) (*21*)					
Fixed and embedded	Brainstem	C57/BL6	2/20	1	588 (NA)
		RIII	3/20	2	535 (5)
		VM	6/20	4	592 (51)
		tg338	10/10	0	NA
Experimental ovine BSE (PG0341/00) (*11*)					
Fixed and embedded	Medulla	RIII	7/20	6	388 (23)
		tg338	5/5	0	NA
Frozen	Medulla	RIII	6/20	0	NA
		tg338	NA	NA	>785¶
Natural bovine BSE (PG0475/05, UK passive surveillance)					
Fixed and embedded	Medulla	C57/BL6	2/20	1	713 (NA)
		RIII	6/20	5	477 (49)
		VM	13/20	2	578 (35)
		tg338	7/10	0	NA
Frozen	Medulla	C57/BL6	7/20	3	535 (76)
		RIII	10/20	9	431 (29)
		VM	16/20	3	568 (31)
		tg338	6/10	0	NA
Natural ovine scrapie (PG2413/98, UK passive surveillance)					
Fixed and embedded	Medulla	RIII	0/20	0	NA
		tg338	5/5	3	97 (2)
Frozen	Medulla	RIII	9/20	8	490 (31)
		tg338	10/10	10	64 (2)

*BSE, bovine spongiform encephalopathy; PrP, host-encoded prion protein; H&E, hematoxylin and eosin; NA, not applicable; UK, United Kingdom. †Animal identification numbers are indicated in parentheses and, where available, references are supplied. ‡Brainstem inocula were prepared by pooling separate samples of brainstem (including medulla, midbrain, and thalamus) retrieved from paraffin wax blocks. §Positive for PrP^Sc^ by using immunohistochemical analysis. ¶Postinoculation time, after which 2 of 10 inoculated mice were still alive at the time of writing; 5 of the 8 mice that died were PrP^Sc^ positive when examined by using immunohistochemical analysis.

Additional controls of fixed and frozen brain tissues from the same source were used to assess the effect of fixation, processing, and retrieval on the biological properties of the TSE agents present. All samples included in this study were from animals showing clinical signs of TSE. These came from animals with confirmed TSE sourced through passive surveillance schemes, with the exception of an ovine BSE case that was produced experimentally (*11*). Because the sampling site of the brain may also affect the infectious titer, in addition to the above parameters we identified a bovine BSE case for which whole frozen brain stem was available. Given the left-right symmetry of PrP^Sc^ distribution, which was verified by IHC analysis of the adjacent rostral and caudal coronal levels of the selected sample, we assumed that titer did not vary substantially on either side of the midline. Therefore, the obex was cut sagitally in half. Half was processed histologically and was subsequently recovered and rehydrated to replicate the process applied in the fixed samples; the other half was kept frozen. Each half was homogenized and inoculated into mice.

Each source was administered to 3 panels of wild-type inbred mice (C57/BL6, RIII, and VM) and a transgenic mouse line (tg388 line was provided by Hubert Laude, Institut National de la Recherche Agronomique, Jouy-en-Josas, France). C57/BL6 and RIII mice share the same PrP sequence (PrP-a), but it is believed that RIII alone could be used to discriminate BSE from other TSEs on the basis of lesion profile (LP) data on first passage (*5*), although this belief has been challenged (*22*). VM mice have a different PrP sequence (PrP-b) and have been used to identify BSE after 2 serial passages on the basis of incubation period (IP) and LP data (*23*). The tg338 mouse line overexpresses an ovine VRQ transgene and has been proved to be susceptible to scrapie (*24**,**25*) and relatively resistant to BSE (*26**,**27*).

Serial passage from the suspected case was initiated only in the VM mouse line because subpassage of BSE in this line gives rise to the mouse-adapted BSE strain 301V, which has a characteristically short IP that can be used to discriminate BSE from scrapie (*23*). The acquired data were compared with an experimental ovine BSE case and with a 301V reference strain that were serially passaged in VM mice in different studies.

The number of mice inoculated with each source varied from 5 to 20 for each mouse line depending on availability of material (Table). Serial passages used 10 mice. Where tissue availability was limited, the mouse lines of choice were RIII and tg338.

### Animal Procedures

Because of the small amount of available material, only intracerebral inoculations (20 μL of 10% of brain homogenate in normal saline) were performed (*22**,**28*). For secondary passage, VM mice were challenged intracerebrally with 20 μL of 1% brain homogenate. Mice were monitored for signs of clinical disease and euthanized either at specified clinical endpoints (*29*) or on the basis of animal welfare justification (intercurrent losses). The brains were removed under sterile conditions by using disposable equipment. Each brain was cut parasagittally, and the smaller fraction was frozen for biochemical analysis or serial bioassay; the larger fraction, which included the midline, was fixed in formal saline and processed for histopathologic and IHC analysis. All animal procedures were performed in compliance with the Animal (Experimental Procedures) Act 1986 under license from the UK Home Office and were approved by the local ethics committee.

### Histopathologic and IHC Analyses

Sections (3 μm thick) of 4 different coronal levels (frontal, thalamic, midbrain, and medulla) were stained with hematoxylin and eosin according to standard methods (*22**,**30*). Positive histologic diagnosis was based on the identification of TSE-related vacuolation. The intensity of vacuolation in 9 gray matter areas was assessed semiquantitatively, and the resultant scores were plotted against the respective brain areas as described (*22**,**30**,**31*).

For IHC evaluation, each section was labeled with a rabbit polyclonal antibody against PrP (Rb486) according to established methods (*22**,**28*). The distribution of different PrP^Sc^ types in the rodent brain at primary passage can provide a means of identifying different strains in wild type (*22**,**28*) and transgenic mice (*32*). By examination of immunolabeled sections, different PrP^Sc^ deposits were identified and their distribution in different neuronatomic brain areas recorded as described (*22**,**28*).

### Western Blotting Analysis

Western blotting (WB) was applied only for PrP-a mice (C57/BL6 and RIII) because PrP-b mice inoculated with either scrapie or BSE produce similar banding profiles and cannot be distinguished by this approach (*33*). Brain homogenates (10% wt/vol for murine samples and 20% wt/vol for ruminant controls) were prepared by using ribosylation tubes (Bio-Rad Laboratories, Hercules, CA, USA). Dilutions of TSE-positive brain homogenates were prepared in known TSE-negative brain homogenates of the same mouse strain. Each brain homogenate was subjected to proteinase K digestion as directed by the manufacturer (Bio-Rad Laboratories) and subsequently prepared for analysis by sodium dodecyl sulfate–polyacrylamide gel electrophoresis and WB according to the TeSeE WB protocol (Bio-Rad Laboratories) with detection of PrP^Sc^ by SHA31, epitope ^148^YEDRYYRE^155^ (proprietary kit reagent) and12B2 (provided by Jan Langeveld, Central Veterinary Institute, Lelystad, the Netherlands), epitope ^93^WGQGG^97^ (0.2 µg/mL) and 12% BisS/Tris (Criterion) acrylamide gels (Bio-Rad Laboratories) in 3-(N-morpholino)propanesulfonic acid buffer. Relative band mass was measured by using Quantity One software (Bio-Rad Laboratories).

## Results

### Attack Rate and IP Analysis

As anticipated (*20*), the use of fixed tissue had a negative effect on attack rate (AR) and IP irrespective of TSE source or mouse line (Table). The most reliable data, suggesting that fixation causes a decrease in titer, are those relating to the bovine BSE, where not only the same source but also the same neuroanatomic region was sampled because of the symmetric distribution of PrP^Sc^ with respect to the midline of the brainstem.

The IP of RIII and VM mice inoculated with material from the goat with suspected BSE were similar to that of the experimental caprine BSE control, but the value of this comparison and that of the other controls was limited because of the generally low ARs observed and the proportion of positive mice that did not progress to show clinical disease. However, because none of the C57/BL6 or tg338 mice inoculated with fixed brain from the goat with suspected BSE showed development of clinical signs of TSE, comparisons of IP in these recipients could not be made.

### LP and Histopathologic Analysis

It is generally accepted that, during first passage, LPs from RIII mice can be used to discriminate BSE from scrapie (*5*), though this principle has been challenged (*22*). An LP is considered to be reliable when >5 clinically and pathologically positive mice contribute to the evaluation (*22**,**28*). Although the ovine and bovine BSE controls fulfilled this requirement, few of the goat-derived samples complied (Table). Therefore, LPs could not be used with any confidence to classify the suspected case.

**Figure 1:**
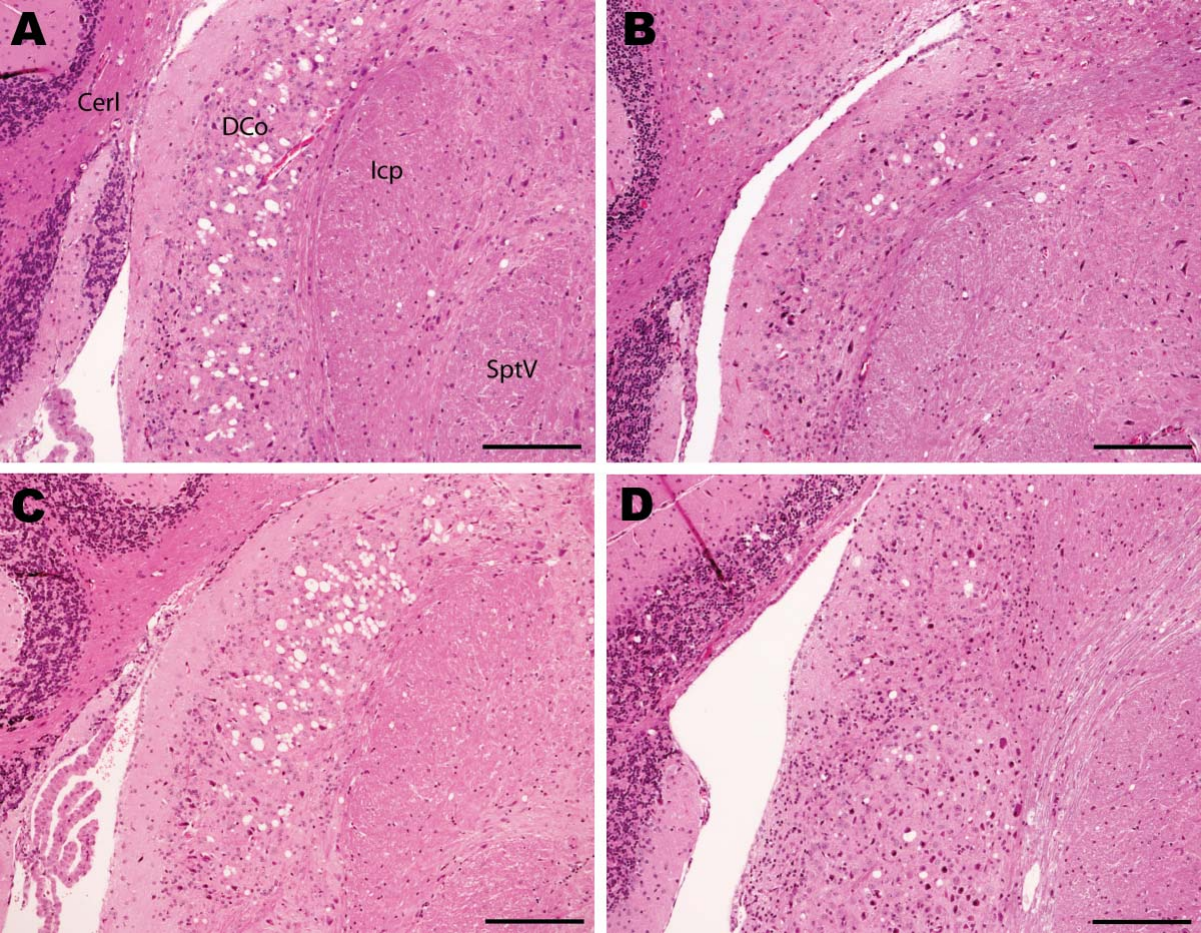
Histopathologic analysis of cochlear nuclei from host-encoded prion protein (PrP)-a mice (C57/BL6) inoculated with (A) fixed material from the suspected case, (B) fixed material from experimental goat bovine spongiform encephalopathy (BSE), (C) unfixed material from experimental sheep BSE, and (D) fixed material from experimental goat scrapie. The BSE-challenged mice (A–C) show confluent vacuolation in the dorsal cochlear nucleus that extends ventrally with increasing lesion severity. Even in mild cases (B) this lesion can be distinguished from the low-frequency randomly dispersed vacuoles observed in scrapie (D). Note the unaffected nature of the lesion between fixed (A and B) and unfixed (C) samples. Cerl, cerebellum; DCo, dorsal cochlear nucleus; Icp, inferior cerebellar peduncle; SptV, spinal tract of the trigeminal nerve. Scale bars = 200 μm.

However, the histopathologic lesions observed individually in all clinically positive mice that were inoculated with material from the caprine BSE-suspected case were consistent with those observed in the known BSE controls, irrespective of whether they came from fixed or frozen tissue, and with previous studies of BSE isolates (*22**,**30*). In addition, they were distinct from the lesions observed in the scrapie controls. These BSE-specific lesions included minimal vacuolation in the ventral midbrain and the cerebellum, characteristic vacuoles in the trigeminal nerve nucleus, and confluent vacuolation in the dorsal cochlear nuclei in PrP-a mice (Figure 1) as described elsewhere (*5**,**22**,**30*).

### IHC Analysis

**Figure 2:**
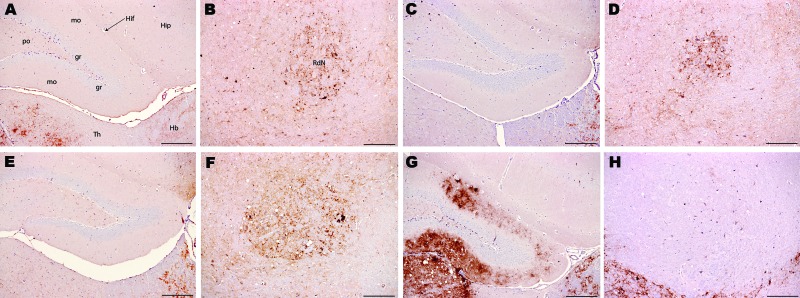
Immunohistochemical analysis of brains of host-encoded prion protein (PrP)-a mice (RIII) inoculated with (A and B) fixed material from the goat with suspected bovine spongiform encephalopathy (BSE), (C and D) fixed material from experimental goat BSE, (E and F) unfixed material from experimental sheep BSE, and (G and H) fixed material from experimental goat scrapie. No PrP^Sc^ was detected in the molecular layer of the dentate gyrus in the suspected case (A) and the BSE controls (C and E); in the scrapie control (G) the same area is heavily affected. In the red nucleus, small PrP^Sc^ aggregates were observed in the suspected case (B) and in the BSE controls (D and F), whereas the same nucleus seem to be unaffected in the scrapie control despite evident PrP^Sc^ deposits in the surrounding area. Hip, hippocampus; Hif, hippocampal fissure; Hb, habenular nuclei; RdN, red nucleus; Th, thalamus; gr, mo, and po, granular, molecular, and polymorph layers, respectively, of the dentate gyrus. Scale bars = 200 μm.

**Figure 3:**
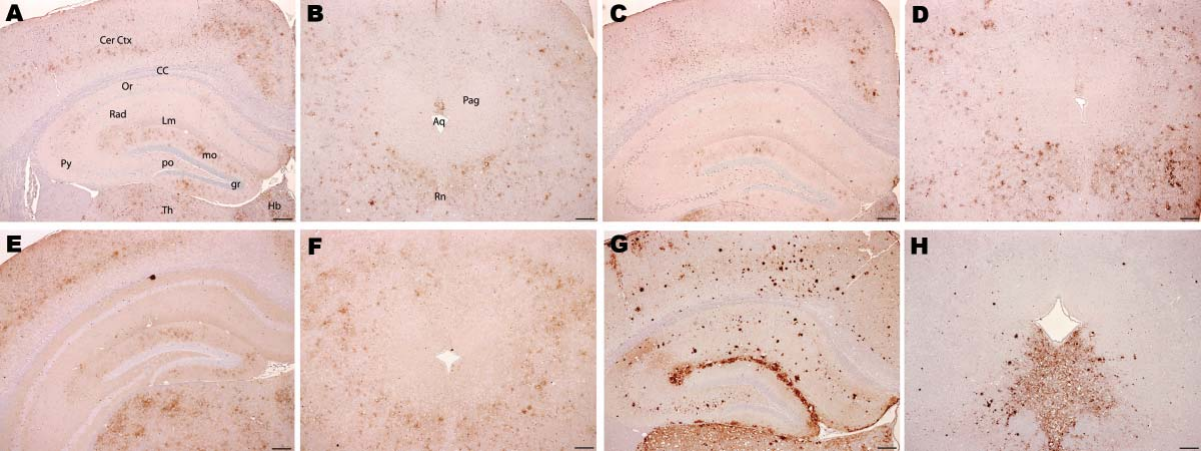
Immunohistochemical analysis of brains of host-encoded prion protein (PrP)-b mice (VM) inoculated with (A and B) fixed material from the goat with suspected case, (C and D) fixed material from experimental goat bovine spongiform encephalopathy (BSE), (E and F) unfixed material from experimental sheep BSE, and (G and H) fixed material from experimental goat scrapie. In thalamus, cerebral cortex, and hippocampus the suspected case (A) and the BSE controls (C and E) showed mainly granular PrP^Sc^ deposits with comparable distribution. In the scrapie control (G), the predominant PrP^Sc^ type was large aggregates and plaques. In the periaqueductal gray matter, mice challenged with the suspected case (B) and with BSE controls (D and F) showed a manifold lower staining intensity of PrP^Sc^ labeling compared with the surrounding area, but the scrapie control (H) showed an intense labeling in the ventral periaqueductal region associated with substantial reduction of labeling in all neighboring areas. Aq, aqueduct; CC, corpus callosum; Cer Ctx, cerebral cortex; Hb, habenular nuclei; Rn, raphe nucleus; Pag, periaqueductal gray; Th, thalamus; Or, Py, Rad, and Lm, oriens, pyramidal cell, radiatum, and lacunosum-moleculare layers, respectively, of the hippocampus; gr, mo, and po, granular, molecular, and polymorph layers, respectively, of the dentate gyrus. Scale bars = 200 μm.

Samples from the goat with suspected BSE and samples from the experimentally BSE-infected goat and experimentally infected sheep generated equivalent PrP^Sc^ distribution patterns in PrP-a mice (Figure 2, panels A–F), which were clearly distinct from the PrP^Sc^ patterns generated by goat scrapie in the same mice (Figure 2, panels G and H). In PrP-b mice, the suspected goat was also indistinguishable from BSE and distinct from scrapie (Figure 3). The BSE-associated PrP^Sc^ distribution pattern was identified in all mice that were inoculated with either frozen or fixed BSE tissues from various sources (Table), suggesting that the histologic processing and suboptimal storage conditions of the archived samples do not alter the biologic properties of the agent. These BSE-related patterns were distinct from the classical scrapie samples that were analyzed here or reported (*22**,**25*).

Clinical-stage TSE (Table) did not develop in any of the BSE-challenged tg338 mice. Therefore, the distribution of spongiform lesions and PrP^Sc^ deposits in tg338 mice in which BSE was diagnosed was limited, and intensity of the labeling was weak. Despite this finding, where PrP^Sc^ distribution was widespread, individual mice challenged with BSE differed qualitatively from those challenged with scrapie (data not shown).

### WB Analysis

**Figure 4:**
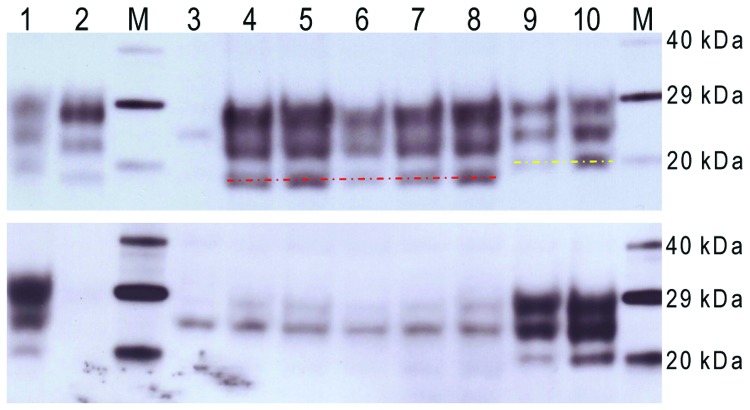
Western blot analysis of a range of murine transmissible spongiform encephalopathy–affected brain homogenates in host-encoded prion protein (PrP)–a (RIII) mice. A) Western blot probed with SHA31, 15-s exposure time. B) Western blot probed with 12B2, 5-min exposure time. M, biotinylated marker; lane 1, ovine scrapie field case; lane 2, bovine spongiform encephalopathy (BSE) field case; lane 3, unchallenged mouse; lane 4, bovine BSE-challenged mouse; lane 5, ovine BSE-challenged mouse; lane 6, caprine BSE-challenged mouse; lanes 7 and 8, mice challenged with suspected case; lane 9, caprine scrapie-challenged mouse; lane 10, ovine scrapie-challenged mouse. Molecular weights are indicated kDa. Red line indicates 19 kDa unglycosylated band; yellow line indicates 20 K unglycosylated band. Identical results were also obtained with C57/BL6 mice.

When examined by WB, brain tissues from PrP-a mice that were inoculated with the sample from the goat with suspected BSE showed PrP^Sc^ bands that were indistinguishable from those of mice inoculated with the various BSE sources (Figure 4). The lower unglycosylated band had a molecular mass of ≈18.8 kDa, and the samples demonstrated lower binding with the 12B2 antibody, confirming that the proteinase K cleavage site was indistinguishable from that of ovine BSE. In contrast, mice inoculated with various scrapie sources demonstrated a 20.1-kDa unglycosylated band and increased reactivity with 12B2 (Figure 4).

### Secondary Passage Data in PrP-b Mice

**Figure 5:**
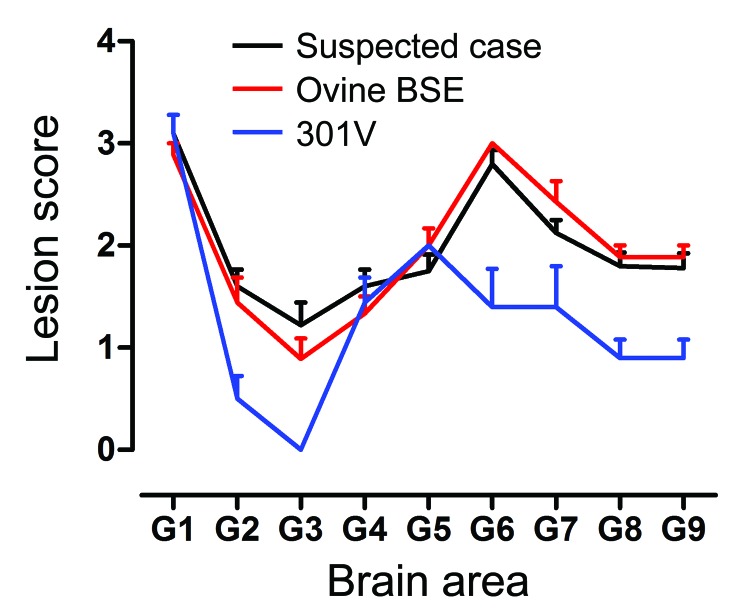
Lesion profiles from VM mice after second passage of the suspected case, serial passage of an ovine bovine spongiform encephalopathy (BSE) source, and a 301V control. Profiles were made on the basis of the lesion score, which is the quantification of transmissible spongiform encephalopathy–specific vacuolation in 9 neuroanatomical gray matter areas: G1, dorsal medulla nuclei; G2, cerebellar cortex of the folia including the granular layer, adjacent to the fourth ventricle; G3, cortex of the superior colliculus; G4, hypothalamus; G5, thalamus; G6, hippocampus; G7, septal nuclei of the paraterminal body; G8, cerebral cortex (at the level of G4 and G5); G9, cerebral cortex (at the level of G7). At least 9 clinically and histopathologically positive mice contributed to each profile. Error bars indicate SEM.

After 1 serial passage in PrP-b (VM) mice, the sample from the goat with suspected BSE generated IP of 128 ± 4 (mean ± SD) days postinoculation similar to serially passaged ovine BSE (109 ± 4) and the 301V mouse-adapted BSE strain (107 ± 6). The comparatively longer IP generated by that goat sample relative to these mouse-adapted BSE isolates is a common observation at second passage; for example, the IP of the serially passaged ovine BSE at second passage was 148 ± 3 days postinoculation. In these mice, the LPs were indistinguishable from those produced by serially passaged experimental ovine BSE and similar to the 301V strain (Figure 5). After serial passage of material from the goat suspected to have BSE in VM mice, the PrP^Sc^ patterns observed were indistinguishable from those induced by other mouse-adapted BSE isolates (data not shown).

## Discussion

We confirmed that the agent responsible for TSE in a UK goat, which was initially reported as scrapie in 1990 and subsequently as suspected BSE in 2006 (*16*), was a BSE agent. This conclusion was based on bioassay of nervous tissue in mice demonstrating similarities of histopathologic lesions, PrP^Sc^ mapping in the brain, and WB of PrP^Sc^ with those of mice inoculated with BSE from various ovine, caprine, and bovine sources.

From a method perspective, the data suggest that AR, IP, and LP are not optimal bioassay parameters for differentiating TSE sources during first passage because they represent mean values derived from a group of animals that have been inoculated with a specific source. Therefore, a substantial number of animals must die of clinical TSE for these parameters to be meaningful. This finding is a limiting factor in instances in which TSE is diagnosed in only a few animals because of low titer, restricted permissiveness of specific TSE strains in certain laboratory animals, or both. These limitations can be overcome by application of IHC and WB to differentiate BSE from scrapie confidently in individual mice on first passage. Use of IHC has shown that different PrP^Sc^ deposits can be identified, and the distribution of each deposit in the brain can be mapped (*22**,**28**,**32*). This approach generates high-resolution data that appear to be specific to individual TSE strains.

The data show that the TSE agents in this study were not altered by the adverse conditions applied to them during histologic procedures. However, titer may decrease, suggesting that the effect of histologic processing is quantitative not qualitative. Therefore, bioassay is a valid approach for identifying BSE in archived histologic material when other techniques are not applicable, as in the current study. Regarding the suitability of different mouse lines for confirming BSE, our data show that any mouse line in which the agent can propagate sufficiently is suitable. An additional requirement at a practical level is the ability to characterize the agent on first passage. In this respect, use of PrP-a mice is preferable because in addition to AR, IP, histopathologic analysis, and PrP^Sc^ patterning, WB can also be applied to diagnose BSE. In contrast, its application in PrP-b mice is less informative (*33*).

These methods can also be applied to analyze bioassay data derived from validated transgenic mouse lines that offer the advantage of higher AR and decreased IP, provided that appropriate transgenic lines are selected and the TSE source and the donor species under investigation are taken into consideration. In this particular instance, our first choices would have been the use of a mouse line overexpressing a bovine transgene in combination with 1 that overexpresses a caprine transgene. At initiation of the study, an established bovinised line was not available to us, and the data generated from the wild-type mice were considered sufficient to identify unequivocally the agent strain. Caprine transgenic mouse lines are still under development and not characterized or widely available. Instead, we used tg338 mice although they show <100% AR and extended IP when inoculated with BSE (*26**,**27*). Our data show that this ovinized line offers a feasible alternative for detecting and differentiating caprine TSEs.

The 2 cases of naturally occurring BSE in small ruminants—the 1 reported here and the 1 identified in France (*15*)—occurred in different countries, during different time periods, and before strict BSE control measures were fully implemented. Therefore, the most likely origin of these 2 cases would be exposure to BSE-contaminated food supplements. Although in France goats constitute 14.3% of the small ruminant population, in the United Kingdom they account for only 0.3% of small ruminants. It is intriguing, therefore, that the only naturally occurring BSE cases in small ruminants in France and particularly in the United Kingdom were detected in goats and not in sheep, although they have also been exposed to contaminated food supplements. A possible explanation could be that goats are generally managed more intensively than sheep and thus might have been exposed to higher doses of the infectious agent because of the more frequent use of concentrates in intensive dairy farming. Similar observations have been reported in cattle, in which the incidence of BSE was significantly higher in dairy herds and in which management is much more intensive than in beef herds (*34*). In the United Kingdom, most of the commercial goat herds are kept for milk production in a typically intensive production system, similar to dairy cattle.

The BSE case we have confirmed was 1 of 26 historic goat samples examined in the United Kingdom collected during 1984–2002 (*16**,**17*). Since 1993, scrapie in goats has been a notifiable disease in the United Kingdom, and since 2005, samples from all suspected cases of TSE in small ruminants are required to be tested for BSE-like features by using WB (*19*). No BSE cases have been identified, although an intermediate case in a goat was reported and is under investigation by bioassay for final resolution (*35**,**36*). This screening of brain samples from all small ruminant cases offers reassurance that BSE is not present in the contemporary small ruminant population. However, application of WB to sheep experimentally co-infected with BSE and scrapie detected only the scrapie agent (*37*). Also, in contrast to BSE, where infectivity is mainly confined to the nervous system, in small ruminants the BSE agent is widely distributed in peripheral tissues and can be transmitted horizontally (*11**,**38*). Therefore, feed ban measures alone would be inadequate to control a BSE outbreak in small ruminants. Also, it would be impossible to prevent BSE from entering the human food chain through consumption of food products derived from small ruminants.

Because TSEs in goats are still a problem, particularly in Mediterranean countries, our data suggest that extensive surveillance and breeding schemes must remain in place to prevent a BSE outbreak in small ruminants and to safeguard public health. This report also highlights several issues regarding the use of mouse bioassay to identify TSE strains. As governing bodies seek confirmation of equivocal cases that are identified worldwide, they must be aware of the limitations, cost, and timescale demands of confirming such cases.
